# A golden age for malaria research and innovation

**DOI:** 10.1186/1475-2875-7-S1-S2

**Published:** 2008-12-11

**Authors:** Awa Marie Coll-Seck

**Affiliations:** 1 Executive Director, Roll Back Malaria Partnership, CH-1211 Geneva 27, Switzerland

## 

The Roll Back Malaria partnership – a consortium of donor and malaria-endemic countries, researchers, policy-makers and private sector enterprises – has helped change the global perspective on malaria. Today, malaria is high on the development agenda, financial resources for countries have increased, more and more success stories in countries are being documented and there is renewed hope that the disease can be beaten. Today, there is new momentum.

In September 2008, the member organizations of the Roll Back Malaria Partnership unveiled the **Global Malaria Action Plan **[1], which, if fully implemented, could save 4.2 million lives by 2015 ...and more beyond.

The Plan is the first single comprehensive blueprint for global malaria control and elimination. It charts the way forward to scale up malaria control in the short term, then eliminate and, eventually, eradicate malaria in the future. It is built on consensus between all partners and it is built on strategies that work. The Plan promotes the use of insecticide-treated nets (particularly those lasting between three to five years) indoor spraying with insecticides, preventive treatment for pregnant women and access to effective treatment – that is, artemisinin-based combination therapy or ACT.

Global in scope, the Plan includes regional approaches, which reach all endemic countries in the world, and it focuses on all species of malaria parasite (*Plasmodium falciparum*, *Plasmodium malariae, Plasmodium ovale *and *Plasmodium vivax*).

For the first time, all stakeholders fighting malaria have at their disposal a single plan agreed by all. But this plan is also a bridge – between good intentions and real action, and between commitments and the delivery and use of interventions in families and villages suffering needlessly from malaria.

The **Global Malaria Action Plan **tells us exactly, region-by-region and year-by-year, what is required and what it will cost.

Despite the efficacy of today's tools, achieving near-term goals of reducing incidence and reducing mortality, as well as the longer-term goals of elimination and eradication, will require new and improved tools that are effective across a variety of settings and populations. Strategies for scaling up, and sustaining control and elimination cannot be discussed without acknowledging the crucial role that research plays in enabling these strategies' success. Lessons learned from the field will feed back into the R&D process, thus informing new research directions. We will also need to generate knowledge to inform policy decisions and improve the use and effectiveness of current and new interventions. The malaria research agenda carries a price tag: USD 750–900 million a year over the next decade for new tools alone. Yet, without this expense malaria cannot be beaten.

If there ever was a golden age for malaria research and innovation, it is now.

Research institutions, public private partnership and joint ventures, have been at the forefront of recent research advances against malaria including vaccine and drug development, supporting local research capacity in Africa and improving delivery of interventions.

Such milestones in scientific and operational research are setting the stage for increased investment in innovative malaria initiatives. At the recent launch of the Global Malaria Action Plan in New York on 25 Sept 2008, The Bill and Melinda Gates Foundation pledged USD 168 million to continued vaccine development and expanding the vaccine R&D pipeline with projects ranging from early-stage laboratory research to advanced clinical testing.

Armed with the Plan's globally agreed research agenda, partners and advocates can now bring more investment to malaria research and foster further dialogue and innovation. The Global Plan requires a long-term commitment: continued funding is essential in both country implementation and R&D to prevent a re-emergence of malaria. However, the investment is worthwhile.

Malaria control is cost effective, saving more lives per dollar spent than interventions for most other diseases. R&D investment today in new and improved interventions can help countries eliminate malaria faster and reduce the need for longer-term R&D costs.

I would like to congratulate the authors of this supplement 'The Scientific Basis of Research for Global Malaria Control' for their comprehensive examination of critical areas that need to be tackled if we are to progress swiftly towards malaria elimination and eventual eradication. From vaccine development, integrated vector control, treatment, confronting economic and financing issues to climate change; these are the very real challenges of today – but, with more investment in research – the accomplishments of tomorrow.

## Competing interests

The author declares that they have no competing interests.
